# Expert Opinions on the Management of Hemophilia A in India: The Role of Emicizumab

**DOI:** 10.7759/cureus.58941

**Published:** 2024-04-24

**Authors:** Naresh Gupta, Anupam Dutta, Bilal Ahmed, Cecil R Ross, Chandrakala S, Gerard Dolan, M J John, Nita Radhakrishnan, Sunita Aggarwal, Tulika Seth, Varun Kaul, Vijay Shah

**Affiliations:** 1 Medicine and Surgery, All India Institute of Medical Sciences, New Delhi, New Delhi, IND; 2 Haematology & Haemophilia, Maulana Azad Medical College, Lok Nayak Hospital, New Delhi, IND; 3 General Medicine, Assam Medical College and Hospital, Dibrugarh, IND; 4 Pathology, Transfusion Medicine & Hemophilia, Government Medical College, Srinagar, IND; 5 Hematology, St. John's Medical College and Hospital, Bangalore, IND; 6 Clinical Haematology, King Edward Memorial Hospital, Mumbai, IND; 7 Haematology, St. Thomas’ Haemophilia Comprehensive Care Centre, Bournemouth, GBR; 8 Clinical Hematology, Hemato-Oncology & Bone Marrow Transplant, Christian Medical College & Hospital, Ludhiana, IND; 9 Hematology and Oncology, Super Speciality Paediatric Hospital and Post Graduate Teaching Institute, Noida, IND; 10 Medicine, Maulana Azad Medical College, New Delhi, IND; 11 Hematology, All India Institute of Medical Sciences, New Delhi, New Delhi, IND; 12 Pediatrics, Guru Gobind Singh Medical College & Hospital, Faridkot, IND; 13 Pediatrics, Nirmal Hospital Pvt. Ltd., Surat, IND

**Keywords:** replacement therapy, inhibitor development, hemophilia a, emicizumab, clotting factor viii, bleeding disorders

## Abstract

Hemophilia A (HA) is a genetic disorder of hemostasis associated with a deficiency or reduced activity of clotting factor VIII (FVIII). This disorder remains unacceptably underdiagnosed in India. Early diagnosis and appropriate management of HA can substantially prevent morbidity and mortality. Currently, HA is managed with regular replacement therapy using standard or extended half-life FVIII concentrates or non-factor drug products. The challenges associated with FVIII concentrates include plateauing of drug effect, issues with its administration and adherence to treatment, breakthrough bleeds, and the development of inhibiting antibodies against administered clotting factors. Emicizumab is a bispecific antibody, launched in India in April 2019, for managing patients with HA. To investigate the role of emicizumab in Indian patients with HA, opinions were sought from 13 eminent hematologists and experts from India on the effectiveness of emicizumab in preventing all bleeds, spontaneous bleeds, perioperative bleeds, and intracranial hemorrhage; resolving target joints; and reducing the rate of hospitalizations and fatality associated with HA in children and adults, with or without inhibitors. The benefits of emicizumab over traditional FVIII concentrates include the subcutaneous route of delivery, less frequent dosing, and a lack of inhibitor development, in addition to providing sustained hemostasis without in-depth monitoring. It is a safe and effective management option for all HA patients, especially for patients with certain archetypes, such as those with inhibitors, those with high annualized bleed rates, those living far away from hemophilia care centers, pediatric patients and infants with intravenous access challenges, and those with a history of life-threatening bleeding events.

## Introduction and background

Hemophilia A (HA) is the most common X-linked hereditary disorder of hemostasis, characterized by a deficiency or reduced activity of clotting factor VIII (FVIII), which is essential for normal blood clotting [1]. HA accounts for approximately 80%-85% of all hemophilia cases [2]. The normal level of FVIII activity is 1 IU/mL of pooled plasma, and the normal activity level ranges between 50% and 100%. Bleeding secondary to minor trauma is seen in mild (FVIII 0.05-0.4 IU/mL; 6%-40%) and moderate (FVIII 0.01-0.05 IU/mL; 2%-5%) deficiencies, but spontaneous bleeding occurs in severe cases (FVIII <0.01 IU/mL; <1%) [1].

According to a report by the Indian Council of Medical Research in 2019, in Western countries, HA was seen in 10 per 100,000 male births, whereas there were an estimated 80,000-100,000 patients with severe HA in India; however, only 19,000 were registered with the Hemophilia Federation of India [3].

An Indian study on 92 patients with hemophilia A and B reported that 61.96% of the patients presented with hemophilia between the age groups of 0-18 years, and most patients (25%) were 11-15 years old. Additionally, severe HA was observed in 63.29% of cases, moderate HA in 22.78% of cases, and mild HA in 13.92% of cases [4].

A diagnostic assessment for hemophilia is conducted when there is a documented family history, excessive bleeding disproportionate to the injury, or an abnormally prolonged activated partial thromboplastin time. In most cases of HA, patients exhibit an extended activated partial thromboplastin time, whereas their hemogram and prothrombin time remain within normal ranges [1]. Iron deficiency anemia is also common among HA patients, and the proportion of patients with iron deficiency anemia increases with the severity of HA. Blood parameters suggestive of anemia may obscure an underlying diagnosis of HA [5].

Regular replacement therapy remains the preferred approach for the prophylactic management of individuals with severe HA and is considered the standard of care [2,6]. According to the World Federation of Hemophilia (WFH), prophylaxis for HA is recommended across countries; however, less intensive prophylaxis may be an option in resource-constrained settings. While on-demand therapy remains rampant, studies have shown that low-dose prophylaxis may be more effective than on-demand therapy in terms of annualized bleed rates (ABRs) [7], as well as disability and cost-effectiveness [8]. The WFH recommends beginning with HA prophylaxis early in life. There are three types of prophylaxis based on when it is initiated and the treatment goals: primary, secondary, and tertiary [9]. Prophylaxis provides sustained hemostatic control, maintaining clotting factor levels within the therapeutic range, for preventing spontaneous and unpredictable bleeding in hemophilia. The benefits of prophylaxis are presented in Table 1 [10].

**Table 1:** Benefits of hemophilia A (HA) prophylaxis Source: Ref. [10]

Benefit	Description
Reduced joint damage and fewer bleeds	Prophylactic treatment prevents spontaneous bleeding into joints, preserving joint function, and reducing the risk of chronic joint disease.
Decreased risk of intracranial hemorrhage	Regular prophylaxis lowers the likelihood of severe bleeding in critical areas, such as the brain, reducing the risk of intracranial hemorrhage.
Long-term benefits and improved QoL	Consistent prophylaxis leads to long-term advantages, including reduced chronic pain, improved joint health, and an overall better QoL so that individuals on prophylaxis experience fewer disruptions to daily activities, enhanced mobility, and improved emotional well-being.
Prevention of hemophilic arthropathy	Prophylaxis helps prevent hemophilic arthropathy, which is characterized by chronic joint inflammation and damage due to repeated bleeding.
Reduced hospitalizations and emergency room visits	Regular prophylactic treatment decreases the need for hospitalizations and emergency room visits related to bleeding episodes.
Prevention of debilitating complications	Prophylaxis helps prevent complications such as muscle and tissue damage, which can occur when bleeding episodes are not promptly addressed.
Enhanced participation in activities	Individuals on prophylaxis can more actively participate in professional, recreational, and educational activities, leading to a more fulfilling and active lifestyle.
Lower healthcare costs in the long run	While the cost of prophylaxis may be substantial, prophylaxis can reduce overall healthcare costs by preventing severe bleeding episodes and associated complications.

Hemophilic arthropathy may be triggered even if a single episode of joint bleeding occurs [11]. An ideal prophylactic treatment for hemophilia aims to minimize bleeding episodes and achieve an ABR of zero, thereby preventing the development of hemophilic arthropathy [12]. In reality, the adoption of prophylactic therapy in India is approximately 4%, compared to 20% in some other developing countries and 80%-90% in developed countries.

Clotting factor concentrates (CFCs), derived from human plasma or produced via recombinant technology from cell cultures [13], are typically used in the prophylaxis of HA. However, their use is limited by their short half-life, need for intravenous administration, and potential for inhibitor formation [10]. Moreover, episodic replacement with CFCs is not advisable in the long term as it does not alter the course of HA related to spontaneous bleeding and associated complications [10]. Up to 30% of patients with severe HA develop anti-FVIII antibodies. In such cases, traditional clotting factor replacement treatments may be less effective [14]. Moreover, testing for inhibitors is not widely or easily available, and once HA patients develop inhibitors, their access to hemophilia care is hampered by the complexity of care with bypassing agents [15]. Furthermore, hematologists’ experience with immune tolerance induction (ITI) in India is limited [15].

The introduction of non-factor therapy (NFT) drug products has revolutionized the approach to the management of HA. These therapies include agents that bypass FVIII inhibition (e.g., activated prothrombin complex concentrates or recombinant factor VIIa), agents that enhance coagulation (e.g., emicizumab), and agents that inhibit anticoagulant pathways (e.g., fitusiran or concizumab) [16,17]. Emicizumab acts as a bridge between factor IX (FIX) and factor X (FX) and enhances coagulation [18]. The WFH recommends that patients with HA initiate home therapy with emicizumab after being trained in the subcutaneous injection technique. Emicizumab is associated with benefits such as subcutaneous administration and avoiding the need for central venous access devices (allowing its use in infants), infrequent dosing of once or twice a month, and not being associated with peaks and troughs in action seen with CFCs. However, breakthrough bleeding must be managed with CFCs or bypassing agents in inhibitor patients [10].

This expert opinion group aimed to discuss the treatment landscape for Indian patients with HA, keeping in mind the global scenario and focusing on emicizumab, which is currently the only licensed nonfactor therapy for HA in India.

## Review

A panel of 13 hematologists and hemophilia experts met in Bangalore, India, in December 2023 to discuss the management of patients with HA and the role of emicizumab in managing Indian patients with HA.

Current challenges in HA management in India

Disability Burden, School/Work Absenteeism, and Economic Burden

Spontaneous or trauma-induced hemorrhagic episodes in HA patients result in a substantial burden when the disease progresses to chronic disability while increasing the risk of premature mortality [19]. Disability occurrence is more common in resource-constrained countries such as India. Of 1,032 patients with HA in a study in Kerala, India, 34 deaths (32.9 deaths/1,000 patients) were reported. Among the patients who died, three patients (8.8%) had inhibitors, and 24 patients (70.6%) had severe hemophilia. More than half of the deaths were due to bleeding (19/34, 55.88%). None of the patients who died were on prophylaxis or home therapy, suggesting that most patients died of preventable causes, and access to prophylactic therapy could have prevented the deaths due to bleeding [20].

Repeated bleeding events among school children can substantially affect the quality of life of these children. In a study conducted on school children with hemophilia in Upper Assam, India, there was a decrease in school absenteeism when the children received either prophylactic (from 15±6 to 4±3) or on-demand (from 10±5 to 3±2) factor replacement therapy, but the decrease was significant only with the prophylactic therapy (p<0.001) [21]. Data from 126 children with bleeding disorders, including hemophilia, from Karnataka, India, showed that joint bleeding (52.9%), especially hemarthrosis of the knee joint, was the most common, followed by psoas bleeds (33.3%), hematemesis, and melena (23.3%) [22]. In this study, bleeding disorders resulted in school absenteeism in 68.25% of the children. Family dysfunction (50.8%); low self-esteem, depression, and parental separation (21.4%); and parental divorce (2.4%) were events reported among the children with HA. In 7.9% of the cases, the mothers of the patients were blamed for the situation and victimized [22].

The level of education attained by the parents of children with HA is also another challenge. For example, the pediatric hemophilia activity list (PedHAL) score is reflective of the functions of standing, kneeling, and sitting among children. In a study on 4- to 14-year-old children with hemophilia from Gujarat, India, the mean PedHAL scores were lower among children whose parents were illiterate (83.1) than among children whose parents had received at least primary schooling (86.55) [23].

Absenteeism at school or work can also affect the financial status of a family, especially if the affected individual is the sole earning member of the family. A cross-sectional study of 160 patients with severe HA from Mumbai, India, linked the poor health status of the patients with their economic burden through increased school/work absenteeism and reduced social competencies, resulting in scholastic backwardness in children and downward social mobility in the career of adults [24].

*Access and Distance to Healthcare Center*s

Access to prophylactic or on-demand therapy at a hemophilia care center is a major driver of bleeding outcomes in HA patients. The previously discussed study on school children in Upper Assam showed a significant reduction in the ABRs of the children after receiving prophylactic treatment (from 37.8±20.0 to 5.8±4.6, p<0.001) and on-demand treatment (from 25.5±19.9 to 8.7±6.2, p=0.006) [21]. The findings of this study indicate that, regardless of the type of HA treatment, access to a hemophilia care center is of prime importance. A retrospective analysis of data gathered over nine years for 211 patients with hemophilia from Punjab, India, showed that 28% of the patients were from rural areas and had to travel a median distance of 63 km (1-214 km) to a healthcare center to receive treatment [25]. Additionally, patients from distant locations find it difficult to travel during active bleeds, emphasizing the need to reduce travel frequency and improve access to prophylaxis to avoid deaths due to preventable causes [20,26]. In the study by Agrawal et al. [20] on patients with HA in Kerala, the number of deaths that occurred at home was three (8.8%), and the distance from the home to the hospital ranged from 17 to 257 km. A cross-sectional study conducted on 101 patients visiting the government medical college in Dehradun, India, found that patients with hemophilia had to travel an average distance of 131.5±83.7 km and took an average of 4.6±3.8 hours to access treatment at their healthcare center [27].

Good access to a hemophilia care center allows the timely management and follow-up of patients with hemophilia, which is extremely important. A 17-year follow-up study showed that patients with hemophilia had hematoma and ecchymosis (29%); prolonged bleeding after an injury or trauma (16%); bleeding from the nose, mouth, or gingiva (12%); severe bleeding following circumcision (5%); gastrointestinal bleeding (5%); and hematuria (1%) [28]. Additionally, 20% of the patients developed arthropathy, and 47% of these patients had to undergo radioisotope synovectomy [28]. Intracranial hemorrhage is also commonly seen in patients with hemophilia. A systematic review of 45 studies involving 54,470 patients showed that neonates with hemophilia have a 33 times higher risk for intracranial hemorrhage than neonates without hemophilia [29].

Target Joint Disability Burden

More than external cuts or wounds, repeated bleeding into joints is considered the main physical concern as it can result in substantial pain, crippling, and permanent joint damage [21]. These joints are called target joints. They are defined as ≥3 hemarthroses occurring in the same joint in the past six months, with recurrent bleeding in weight-bearing joints (knees, ankles, and elbows), leading to progressive damage of the joints, and hemophilic arthropathy characterized by bony changes, loss of joint space, synovial hypertrophy, and damage to the cartilage [30]. A cross-sectional analysis conducted in West Bengal, India, found that 54.7% of the 201 patients with hemophilia had target joints (no axial deformity, 85.6%; Grade I axial deformity, 13.4%; Grade II axial deformity of the ankle joint, 1%) [30]. In another study conducted in Uttar Pradesh, joint involvement was present in 77% of the patients with hemophilia (knee affected in 57.1%), compromised joint movement in 76.6% due to joint swelling, and joint bleeding in 15.6% [31].

**Figure 1:**
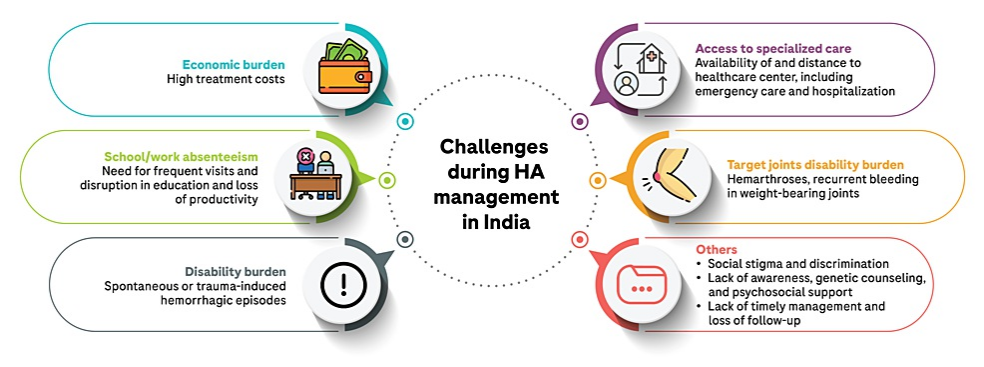
Challenges during HA management in India HA: Hemophilia A Original illustration

To further emphasize the orthopedic burden associated with HA, the HAEMOcare study, which was the first multicenter epidemiological study conducted across six low- and middle-income countries, including India, identified inadequate access to a hemophilia care center and high bleeding rates as major drivers of morbidity due to orthopedic burden among HA patients [32]. In that study, a majority (70%) of the HA patients reported pain/discomfort and impaired mobility as factors that negatively affected their quality of life. Figure 1 summarizes the commonly faced challenges during HA management in India. 

Need for emicizumab prophylaxis

Previously, the prophylaxis protocol for patients with severe HA aimed to achieve ≥1% trough levels of FVIII. However, HA patients with ≥1% FVIII encounter spontaneous bleeding and are at risk of injuries during daily activities. Additionally, these levels of FVIII are inadequate to prevent arthropathy [33].

Subsequently, higher trough levels were achieved with different regimens or newer clotting products with extended half-life (EHL), but the problem of breakthrough spontaneous joint bleeds and damage remained. This was followed by the introduction of NFT as a different treatment modality.

Emicizumab is a bispecific antibody that contains two antigen-binding domains: one recognizes FIX/factor IXa (FIXa) and the other recognizes its epitope on FX/factor Xa (FXa). It acts as a bridge between FIXa and FX, bringing the enzyme and substrate into proximity. This interaction enhances FX activation through FIXa, ultimately supporting the formation of thrombin by FXa and raising a robust hemostatic response [18]. Expert opinions on the need for emicizumab prophylaxis are mentioned below:

“Only 40% of the patients on either standard half-life or extended half-life FVIII were bleed-free.”

“Challenges associated with factor VIII concentrates included plateauing of the drug effect, issues with drug administration and adherence, breakthrough bleeding, venous access challenges, and the development of inhibitors.”

“All treatments thus far have targeted the factor VIII pathway. However, emicizumab has a different mechanism of action. It mimics the action of factor VIII. No other drug has been able to manipulate the coagulation system and reduce bleeding at par with emicizumab.”

“Emicizumab has shown a remarkable reduction in ABR (even in inhibitor patients) with a greater number of patients who were bleed-free as compared to those on half-life and extended half-life concentrates of factor VIII.”

“Emicizumab has obtained early approval in the UK for managing inhibitor patients. It has been effective in reducing the number of target joints with barely any production of neutralizing antibodies. Adverse effects were observed only in patients concurrently receiving activated prothrombin complex concentrates (aPCC).”

“There is a need to understand the role of emicizumab in managing patients with and without inhibitors and in patients with different bleeding phenotypes. Customizing drugs as per the disease phenotype may be associated with a greater success rate.”

“Currently, there are multiple therapeutic options, and there is a need to prioritize these options for patients on a case-by-case basis considering various patient-related factors including the patient’s age and access to a hemophilia care facility.”

“Adoption of objective criteria to identify patients who can benefit from these therapies will help reduce inequities in hemophilia care.”

Emicizumab in India

Emicizumab was launched in India in April 2019 as a treatment option for the management of HA in patients with FVIII inhibitors. It was the first agent with a subcutaneous mode of delivery to remarkably reduce bleeds [34].

The results of various landmark clinical trials on emicizumab prophylaxis conducted globally are summarized in Table 2 [35-50].

**Table 2:** Trials on the effectiveness and safety of prophylactic emicizumab in the management of patients with HA Sources: Refs. [35-50] ABR: Annualized bleeding rate; ADA: Anti-drug antibody; aPCC: Activated prothrombin complex concentrate; CVAD: Central venous access devices; EmiPref: Emicizumab preference survey; FVIII: Factor VIII; HA: Hemophilia A; Haem-A-QoL: Hemophilia-specific health-related quality of life questionnaire; HRQoL: Health-related quality of life; PH: Physical health; SD: Standard deviation; TMA: Thrombotic microangiopathy; TS: Treatment satisfaction

Authors, year	Name of the trial and study design	Study population	Intervention	Primary outcome
In patients with inhibitors				
Oldenburg et al. [35], 2017	HAVEN 1 A phase 3, open-label, multicenter, randomized trial	109 male patients ≥12 years of age with HA with inhibitors	Patients with HA who were on priorly managed with episodic bypassing agents were administered prophylactic emicizumab (group A) or were not provided with prophylactic therapy (group B). Group C consisted of patients who were previously on prophylactic bypassing agents and were later administered prophylactic emicizumab.	The ABR in group A patients was 2.9 events as compared to 23.3 in group B patients (p<0.001). While 22 patients from group A reported no bleeding events, only 1 patient from group B reported the same. A 79% lower bleeding rate was observed in the patients in group C as compared to the rate of bleeding when they were on prophylactic bypassing agents (p<0.001). The most common adverse event observed was a reaction at the injection site (15%). Thrombosis and TMA were recorded in 2 patients each. These patients had received aPCC for breakthrough bleeds. There was no formation of antidrug antibodies. This study demonstrated that emicizumab helped reduce the ABR by 87% as compared to management with episodic bypassing agents in patients with inhibitors.
Young et al. [36], 2019	HAVEN 2 A phase 3, multicenter, open-label trial.	85 patients <12 years of age with HA with inhibitors	Patients on prior prophylactic or episodic bypassing agents were administered 1.5 mg/kg subcutaneous emicizumab weekly in group A, 3 mg/kg every 2 weeks in group B, and 6 mg/kg every 4 weeks in group C.	The results demonstrated that a once-weekly dose of prophylactic emicizumab administered subcutaneously reduced the bleeding rate effectively, with most participants (77%) having no treated bleeding events. Additionally, all the target joints evaluated resolved while the patient was on emicizumab prophylaxis. A 99% decrease in bleeding rate was seen with emicizumab as compared to prophylaxis with a bypassing agent and the efficacy was maintained even when the frequency of dosing was reduced. Irrespective of the dosing schedule, the patients experienced ≤3 treated bleeding events. Of all CVADs performed, 17 (81%) were performed without prior administration of a bypassing agent and were associated with just one treated bleed. This was the first study that reported resolved target joints in patients with inhibitors. Furthermore, no thrombosis or TMA was observed, and emicizumab prophylaxis was associated with a favorable safety profile.
Mahlangu et al., 2018	HAVEN 3 A phase 3, multicenter, open-label, randomized trial	152 patients with HA, ≥12 years of age, without inhibitors	The patients in group A received 1.5 mg/kg body weight per week of maintenance dose of emicizumab, group B received 3.0 mg/kg every 2 weeks, group C did not receive any prophylactic therapy, and group D previously on FVIII prophylaxis received a maintenance dose of 1.5 mg/kg emicizumab per week.	An ABR of 1.5 events was observed in group A, 1.3 in group B, and 38.2 in group C. The bleeding rate was reduced by 96% in group A as compared to group C (p<0.001) and by 97% in group B as compared to group C (p<0.001). There were no bleeding events in 56% of the group A patients and 60% of the group B patients. In contrast, all patients in group C had bleeding events. Additionally, the ABR in the group D patients was 1.6, and 56% of the patients encountered zero bleeding events. No thrombotic events, TMA, or death of the patient occurred. Patients on emicizumab did not develop any new FVIII inhibitors. The study concluded that emicizumab when administered every week or every 2 weeks was associated with significantly lesser bleeding rates when compared to no prophylaxis.
In patients without inhibitors				
Négrier et al. [50], 2023	HAVEN-6 A phase 3, multicenter, open-label, single-arm study	72 patients with mild or moderate HA without FVIII inhibitors	Subcutaneous emicizumab was initiated at a dose of 3 mg/kg of body weight once weekly for weeks. This was followed by a maintenance dose of the patient’s choice, either 1.5 mg/kg every 2 weeks or 6 mg/kg every 4 weeks.	Exploratory analyses showed that in patients with moderate HA, the model-based ABR (95% CI) decreased from 6.0 (4.33–8.22) to 2.2 (1.57–3.20), whereas, in patients with mild HA, it decreased from 20.2 (8.11–50.27) to 2.4 (1.28–4.53). Additionally, 21 patients underwent surgery during the trial period; 18 of them underwent 33 minor surgeries, and 3 underwent one major surgery. Of the 33 minor surgeries, 13 were performed without additional prophylaxis for HA, 20 were performed with prophylactic therapy (14 with only FVIII, 3 with tranexamic acid with FVIII, and 3 with only tranexamic acid). Of 52 patients ≥12 years of age who answered the EmiPref questionnaire, 50 patients preferred emicizumab to the previous therapy, 1 preferred the previous therapy, and 1 expressed having no preference. Of 28 caregivers, 24 preferred emicizumab, 1 preferred the child’s previous therapy, and 3 expressed having no preference for one over the other. The mean (SD) change in target joints was -2.11 (4.66) and 20 out of 21 patients who had target joints had <3 bleeds over 52 weeks, thus, meeting the criteria of target joint resolution. Although 2 patients developed ADAs, the ADAs did not affect the pharmacokinetics of emicizumab. The results demonstrated the favorable benefit-risk profile of the drug in patients with mild and moderate HA without FVIII inhibitors.
Effectiveness of once-a-month dosing				
Pipe et al. [44], 2019	HAVEN-4 A phase 3, multicenter, open-label, non-randomized study with a 2-stage design	41 patients ≥12 years of age with severe HA or HA with inhibitors and undergoing treatment with bypassing agents or FVIII concentrates	All patients were administered subcutaneous emicizumab 6 mg/kg every 4 weeks over a period of 24 weeks or greater. In the expansion cohort, prior to the 6 mg/kg every 4 weeks dose, four loading doses were given in a dose of 3 mg/kg once a week.	The model-based ABR was 2.4 (1.4–4.3), the median ABR was 0 (0.0–2.1), 23 (56.1%) patients had no treated bleeds and 37 (90%) patients reported 0–3 treated bleeds. Of the treated bleeds, 75% were traumatic and 26% were spontaneous. Furthermore, in 35 (85%) patients, there were no treated target joint bleeds. The results of the study showed that emicizumab given every 4 weeks consistently controlled bleeding, irrespective of FVIII inhibitor status. Additionally, emicizumab demonstrated efficacy in preventing spontaneous bleeds.
In the management of patients with HA in the Asia-Pacific region				
Yang et al. [39], 2022	HAVEN-5 A phase 3, multicenter, open-label, randomized clinical trial	70 patients ≥12 years of age with severe HA or HA with inhibitors with ≥5 bleeds and on episodic bypassing agents or FVIII concentrates from the Asia-Pacific region	Patients in all arms were given a loading dose of 3 mg/kg emicizumab once a week for 4 weeks followed by maintenance therapy. The patients in arm A were administered 1.5 mg/kg emicizumab once a week, arm B patients were given 6 mg/kg emicizumab every 4 weeks, and patients in arm C received no prophylactic therapy.	In arm A, the median efficacy period was 43.7 (36.14–48.43) weeks, for arm B it was 46.1 (36.71–49.29) weeks, and for arm C, it was 24.0 (24.00–24.49) weeks. The model-based ABR for treated bleeds for arm A was 1.0 (0.53–1.85), for arm B was 1.0 (0.50–1.84) and for arm C was 27.0 (13.29–54.91). A statistically significant reduction in ABR by 96% was observed in arm A and arm B as compared to arm C (p<0.0001). Zero bleeds were seen in 65.5% of patients in arm A, 55.6% of patients in arm B, and 17.1% of patients in arm C. The median ABR was zero for spontaneous bleeding, treated joint bleeding, and treated targeted joint bleeding for arms A and B. In arm A, 82.8% of patients had zero target joint bleeds; in arm B, this was seen in 70.4% of patients as compared to 28.6% of patients in arm C. Emicizumab demonstrated greater efficacy than no prophylaxis in patients with and without FVIII inhibitors. There were no deaths, thrombotic events, or TMA reported. Therefore, emicizumab administered either once weekly or every 4 weeks significantly decreased ABRs in patients with HA from the Asia-Pacific region and had a favorable safety profile.
Liu et al. [40], 2022	Retrospective, observational, real-world study	13 pediatric patients with severe or moderate HA irrespective of inhibitor status	Data on demographic features, diagnosis, history of inhibitors, prophylactic treatment regimen and history of bleeding events including traumatic bleeds, joint bleeds, treated bleeds and all bleeds from 6 months prior to emicizumab administration was collected. Emicizumab was administered at a loading dose of 3 mg/kg every week for the first week followed by a maintenance dose of 1.5 mg/kg weekly or 3 mg/kg every 2 weeks, or 6 mg/kg every 4 weeks.	A total of 11 events of bleeding were reported in the patients while on emicizumab, 6 of these were untreated events of bleeding. After an average of 17.54 (6–26) months of switching to emicizumab the ABR reduced from 4 (0–18) to 0.5 (0–4), the joint bleeds from 1.0 (0–12) to 0 (0–1) and spontaneous bleeds from 2.0 (0–18) to 0 (0–1) as compared to the bleeding rates that were observed 24 weeks before emicizumab (p<0.01). Additionally, the number of patients were zero bleeds increased from 7% to 46% after switching to emicizumab. This real-world study showed a remarkable improvement in the number of bleeds in pediatric patients, with or without FVIII inhibitors, on emicizumab.
Long-term outcomes				
Callaghan et al. [41], 2021	HAVEN 1–4 pooled data	Pediatric and adult patients with HA, with or without FVIII inhibitors enrolled in HAVEN 1–4 studies	Data from 400 patients for the efficacy group and from 399 patients in the safety arm were descriptively analyzed.	For the median efficacy period of 120.4 (89.0–164.4) weeks, the model-based ABR was 1.4 (1.1–1.7). Across the studies, the mean ABR for treated bleeds reduced over successive 24-week treatment intervals, and the median ABR for treated bleeds remained 0 for the entire study period. Additionally, 97.6% of the patients on emicizumab experienced ≤3 treated bleeds and 82.4% of patients reported 0 treated events of bleeding. ABRs for treated bleeds were not affected by the inhibitor status of the patient. The model-based ABR for treated spontaneous bleeds was 0.6 (0.4–0.8) and the median ABR for treated spontaneous bleeds remained 0 across 24-week time intervals. The model-based ABR for joint bleeds was 0.9 (0.7–1.2) and a total of 98.2% of patients, at weeks 121–144, reported ≤3 treated joint bleeds. The model-based ABR for treated target joint bleeds was 0.5 (0.4–0.7) during the entire study period and during weeks 121–144, 94.1% of the patients had 0 treated target bleeds and 99.4% reported ≤3 treated target bleeds. There were 3 incidences of TMA and 2 events of thrombosis associated with the concomitant use of aPCC. There was one event each of myocardial infarction and venous device occlusion. The analysis of the pooled data showed that in the 970.3 patient-years of exposure, prophylaxis with emicizumab was well tolerated and associated with low bleed rates in patients with HA across age groups and in patients with and without FVIII inhibitors.
Skinner et al. [42], 2021	HAVEN 3–4 pooled data	Patients ≥18 years old	Data from 176 evaluable patients from HAVEN 3–4 on HRQoL assessed using the Haem-A-QoL questionnaire was analyzed.	Of the 176 patients evaluated, 55% had received episodic therapy previously and 45% had been on prophylactic treatment, 51% reported ≥9 bleeds in the previous 24 weeks, and 70% had ≥1 target joint. The mean Haem-A-QoL TS and TS improved following the initiation of emicizumab therapy. A clinically meaningful improvement in PH scores by ≥10 points was seen in 54% of patients by week 73. Patients with poorer HRQoL, before the initiation of emicizumab treatment, reported the greatest improvement in PH scores and reductions in work absenteeism.
Jiménez-Yuste et al. [47], 2022	STASEY A phase 3b, multicenter, single-arm study	Patients aged ≥12 years with congenital HA and FVIII inhibitors	193 patients received prophylactic emicizumab once a week.	The most common adverse effect observed was arthralgia (17.1% of patients), whereas the most common treatment-related adverse event was injection-site reaction (9.8% of patients). Two patients died, one due to polytrauma and fatal head injuries, the other due to abdominal compartment syndrome. Neither of the deaths was related to emicizumab. There were two events of thromboembolism (localized clot after tooth extraction and myocardial infarction), which were also unrelated to the drug. Patients with HA and FVIII inhibitors had a similar safety profile in the STASEY study as was observed in the HAVEN studies.
Shima et al. [43], 2020	A multicenter, open-label, non-randomized, phase 1 study	18 Japanese patients ≥12 years of age with severe HA with or without inhibitors	Emicizumab was initially given at a maintenance dose of 0.3, 1, or 3 mg/kg via the subcutaneous route once a week and was later switched to a maintenance dose of 1.5 mg/kg.	The patients were followed up for a period of up to 5.8 years. The adverse events reported were mild and unrelated to the drug. The median ABR was 1.25 with the 0.3 mg/kg dose, 0.83 with the 1 mg/kg dose, and 0.22 with the 3 mg/kg dosing of emicizumab. Regarding the patient perception of the drug, all the patients mentioned that the bleeding symptom and the time until the bleeding stopped improved, except one patient who mentioned that it slightly improved. Additionally, most patients stated that their daily lives and feelings either improved or slightly improved due to emicizumab prophylaxis. All patients but one mentioned that anxiety also improved or slightly improved after initiation of emicizumab. Therefore, the study results demonstrated that emicizumab was safe when administered for a long term (5.8 years).
Role in infants and children				
Pipe et al. [44], 2023	Primary analysis of HAVEN 7 A phase 3b, multicenter, open-label, single-arm trial	Infants ≤12 months of age with severe congenital HA without FVIII inhibitors	A maintenance dose of emicizumab 3 mg/kg every 2 weeks for 52 weeks continued during the 7-year follow-up.	The model-based ABR for treated bleeds was 0.4 (0.30–0.63) with 54.5% of patients with zero treated bleeds. The treated bleeds were due to trauma. Emicizumab-related adverse events (injection site reactions) were seen in 16.4% of patients. There were no incidences of thromboembolism, TMA, development of ADAs, death, or changes in treatment due to adverse events. In infants with HA, early prophylaxis can reduce potentially life-threatening bleeding and long-term joint function. Furthermore, the subcutaneous route of delivery made it easy to administer prophylactic emicizumab in infants.
Role in perioperative hemostasis				
Kruse-Jarres et al. [48], 2017	Analysis from surgical analysis from HAVEN 1 and interim analysis of HAVEN 2	22 patients who underwent surgical procedures	Patients from HAVEN 1 and 2 on prophylactic emicizumab therapy.	Of 29 surgical procedures, 20 (69%) were managed without prophylactic bypassing agents. Out of these 20 surgeries, 14 (70%) did not result in post-operative bleeding, and 6 (30%) resulted in post-operative bleeding (2 of these were treated with bypassing agents). The analysis reports showed that most patients with HA, with inhibitors who underwent surgery (mostly minor), rarely needed bypassing agents to manage perioperative bleeding.
Kruse-Jarres et al. [45], 2023	HAVEN 1-4 pooled data	233 surgeries carried out in the HAVEN 1–4 trials	Patients from HAVEN 1–4 on prophylactic emicizumab therapy.	Of the 233 surgeries that patients underwent during the HAVEN 1–4 trials, 215 were minor surgeries in 115 patients with HA (64 with inhibitors) and 18 were major in 18 patients with HA (10 with inhibitors). Out of the 215 minor surgeries, 141 (65.6%) did not require additional prophylactic FVIII concentrate to manage bleeding, and 121 (85.8%) surgeries were not associated with any incidence of postoperative bleeding. Among the 18 major surgeries, 15 (80.0%) encountered no postoperative or intraoperative bleeds. The results from the analysis indicated that major and minor surgeries can be safely performed in patients with HA on emicizumab prophylactic therapy.
Castaman et al. [49], 2021	Final analysis of the data from the STASEY study	46 patients from the STASEY study	Patients from the STASEY study who underwent surgery while on emicizumab prophylaxis.	Of the 46 patients who underwent surgeries, 37 patients had 56 minor surgeries and 24 (42.9%) of these were managed with the addition of prophylactic medication. Out of these 24 surgeries, 11 (45.8%) were associated with postoperative bleeds, and 6 out of 11 (54.5%) were treated. Out of the surgeries managed without any additional prophylactic medications, 15 out of 32 (46.95) were associated with postoperative bleeds, and 5 out of these 15 (33.3%) were treated. Out of the 22 major surgeries that 13 patients underwent, 18 (81.8%) were managed with additional prophylaxis and 4 were managed without additional prophylaxis. Therefore, in the STASEY study, most patients underwent minor surgery without the need for additional prophylactic FVIII concentrate and did not have postoperative treated bleeds.
Lewandowska et al. [46], 2021	Real-world experience	30 surgeries in patients with HA with or without inhibitors	Data on demographic characteristics, medical history, disease characteristics, management, and procedure-related bleeding was collected.	A total of 22 patients underwent 25 surgeries, 20 were minor, and 5 were major. Out of the 20 minor surgeries, 9 were performed with no additional prophylactic agent other than emicizumab, and 4 needed an additional coagulation factor. Additional coagulation factors were used in all major surgeries and there was one bleed reported. No thrombotic events, major bleeds, or deaths were reported. The results of the study demonstrate that a variety of surgeries can be performed safely in patients with HA on prophylactic emicizumab and no risk of thrombosis was observed with the use of additional hemostatic agents with prophylactic emicizumab.

Emicizumab trough levels of 3.8-9.8 µg/mL, equivalent to FVIII levels of 1%-3%, have been achieved with a monthly dose of prophylactic emicizumab at 1.1-1.6 mg/kg without a loading dose. To put this in context, lower trough levels of FVIII (i.e., below 1%) substantially increase the risk of joint bleeds, and there is a high chance of sustaining irreversible joint damage with as few as three episodes of joint bleeds [51]. Therefore, to achieve a zero-bleed strategy, a prophylactic agent must achieve trough levels equivalent to 1%-3% while considering the patients’ bleeding pattern, condition of the musculoskeletal system, physical activity, and baseline levels of coagulation factors [51].

Low-dose emicizumab was effective in patients with HA, with or without inhibitors, who performed low bleeding risk activities [52]. A recent single-center, pilot study conducted in India on a small group of eight patients with HA showed that emicizumab administration at a low dose of 0.84-2.6 mg/kg once every four weeks was effective in reducing bleed rates throughout the follow-up period of one year; there was no evidence of reported bleeding events during the prophylaxis period [53]. In this study, the patients were exposed to low-impact physical activities such as swimming, cycling, and walking. However, low-dose prophylaxis does not align with the recommended label usage.

On examining the clinical course of Indian children with high-titer inhibitors and severe HA who were switched from on-demand bypassing agent therapy to emicizumab prophylaxis, no bleeds were reported after prophylactic therapy with emicizumab was initiated. No adverse events, except occasional pain at the injection site, were observed. Due to the bleed rate being reduced to 0%, the joint status in these patients improved, and patients showed an improvement in the PedHAL score as early as four weeks after emicizumab prophylaxis was initiated. Emicizumab prophylaxis in these patients resulted in a clinical reversal of arthropathy, an improvement in health-related QoL, and reduced incidence of spontaneous bleeding [54]. Long-term trials from India with large sample sizes are scarce, and experience with emicizumab prophylaxis from India is largely anecdotal. Expert opinions on the need for additional research on emicizumab prophylaxis in India are mentioned below:

“Given that more than 1000 patients in India are currently on prophylactic emicizumab, the experts agreed that there is a need to collect real-world data on the effectiveness of emicizumab in India.”

Cost-effectiveness of emicizumab prophylaxis and the need for health economic outcomes research (HEOR) studies

**Figure 2:**
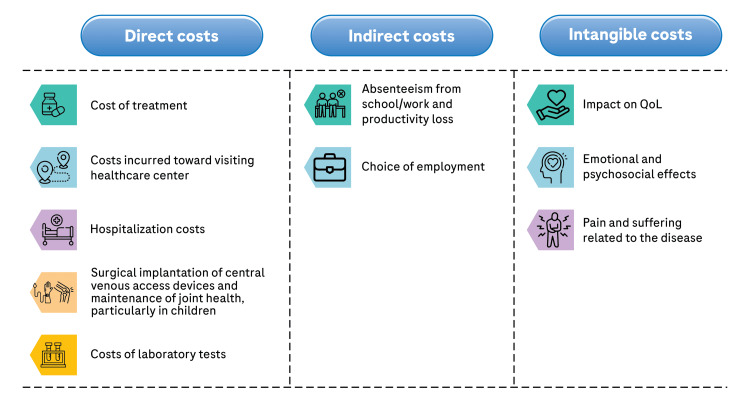
Direct, indirect, and intangible costs associated with HA management HA: Hemophilia A; QoL: Quality of life Original illustration

HA is a lifelong condition requiring regular treatment, with individual costs depending on disease severity and treatment regimen. Direct costs, such as medication and medical procedures, form the bulk of expenses, while indirect costs stem from decreased productivity and increased absenteeism due to the condition and its treatment. Additionally, intangible costs encompass the impact on quality of life and emotional well-being, including the pain and suffering associated with the disease [55]. Costs related to HA management are shown in Figure 2. Emicizumab reduces several patient-borne costs as compared to therapy with FVIII concentrate.

The cost of the drug has been considered the most important barrier to its uptake [56]. However, modeling studies have reported the cost-effectiveness of emicizumab prophylaxis over FVIII prophylaxis and over emicizumab treatment, low-dose prophylaxis, intermediate-dose prophylaxis, and high-dose prophylaxis [8,57].

In an economic model developed to predict the short-and long-term clinical and economic outcomes of prophylactic emicizumab compared to short-acting recombinant FVIII in patients with HA in the US, prophylactic emicizumab had 33% lower total direct costs (cost of treatment, medical costs, and cost in managing severe adverse events) and 56% lower indirect costs (loss of productivity as a result of unemployment or part-time work) than FVIII prophylaxis over a lifetime [58].

Another decision model developed from the viewpoint of a US payer showed that patients with severe HA, starting at the age of one year, with no prior FVIII exposure and initiating prophylaxis with FVIII or emicizumab, had to pay 34% lower for emicizumab prophylaxis than with for FVIII. This difference in costs incurred was due to early inhibitor development (four months with FVIII and 162 months with emicizumab) and a switch to a bypassing agent [57]. While the results from this model demonstrate the cost-effectiveness of emicizumab in the management of previously untreated patients with severe HA compared to FVIII prophylaxis (current standard of care), data related to the Indian context are scarce.

A Markov model economic analysis in non-inhibitor patients with severe HA from India showed that emicizumab prophylaxis was cost-effective compared to high-dose prophylaxis (7,125 IU/kg/year) with an incremental cost-effectiveness ratio per quality-adjusted life year of INR 27,869. Emicizumab prophylaxis had a 49.4% and 94.7% probability of being cost-effective when the willingness-to-pay threshold was two and three times the per capita gross domestic product, respectively [8]. The clinical, humanistic, and economic benefits of emicizumab prophylaxis outweigh the other treatment options and make it the treatment of choice, particularly in resource-constrained settings. Expert opinions on the cost-effectiveness of emicizumab are mentioned below:

“The main challenge to the use of non-factor therapies in resource-constrained settings remains the cost of therapy.”

“The benefits of emicizumab override the cost of the drug. It is effective as a prophylactic/maintenance therapy in patients of all ages and patients with systemic diseases such as hepatic disease, and renal problems.”

“Emicizumab has been well received in India, however, more data from India could prompt the government to increase the funds for treating HA and in turn the number of patients who can access the drug would increase. Currently, the Employees’ State Insurance Corporation has funds for the procurement of the drug and fully supports its use. The labor law of the country does not allow denying the drug to patients belonging to the lower socio-economic strata.”

Safety

Thrombotic microangiopathy (TMA), as a complication of emicizumab, was first reported in the HAVEN 1 trial. This adverse event was reported in three of the 109 patients on the drug and was considered a chance occurrence unrelated to the drug. An analysis of patients with TMA demonstrated that these patients were prescribed activated prothrombin concentrate (aPCC) >100 U/kg per day to manage acute bleeding episodes. Factors IX/IXa and X/Xa are present in aPCC, and these factors act as substrates for emicizumab. Thrombotic complications are due to an excess of FIXa, leading to an uncontrolled production of thrombin. However, TMA in these patients resolved rapidly after aPCC was discontinued. Additionally, an inhibitor patient from the HAVEN 1 trial died due to rectal bleeding. The HAVEN 1 trial investigator reported that the TMA was resolving at the time of death and the cause of death was unrelated to the drug [35,59].

As with all drugs, there is a potential for the development of anti-drug antibodies (ADAs) against emicizumab. These ADAs can reduce the treatment response to emicizumab and can lead to a loss of efficacy by either the inhibition of drug activity or the acceleration of drug clearance. A study analyzed the incidence of ADAs to emicizumab and the effect of ADAs on the pharmacodynamics, pharmacokinetics, safety, and efficacy of the drug from the results of seven phase 3 or 3b clinical trials. The analysis of 688 patients with HA on emicizumab revealed that 5.1% of the participants (n=31) developed ADAs, 0.6% of participants (n=4) demonstrated decreased concentration of emicizumab, and only 0.1% of participants (n=1) discontinued the drug due to a loss in its efficacy. Therefore, the immunogenicity of emicizumab as reported based on the results of phase 3 clinical trials was found to be low, and routine surveillance is not warranted. However, if the efficacy of the drug is reduced, the treating physician needs to evaluate the patient for ADAs [60].

Clinicians must also consider assay interference when ordering tests for FVIII activity or inhibitor levels in patients using emicizumab. As the drug alters coagulation, it is also expected to influence tests based on intrinsic clotting, such as the activated partial thromboplastin time [1]. Clotting-based tests may inaccurately elevate FVIII activity levels, potentially leading to a false sense of security and increased risk of bleeding in patients [61]. Expert opinions on the safety of emicizumab are mentioned below:

“In the UK, patients with leukemia and cerebral aneurysm had been allowed to use emicizumab as per the compassionate use program. The cause of death in patients with leukemia or cerebral aneurysm was the disease and was unrelated to the use of emicizumab. Rather, fatality in many patients with HA has been attributed to inadequate access to drugs.”

“When non-factor therapies were introduced, there was a concern regarding TMA. TMA was seen in patients who were concomitantly on aPCC therapy. However, the use of aPCC is now declining. Furthermore, thrombotic events related to emicizumab have not been associated with deaths as such events are easily managed with early intervention.”

“Before any biologic is approved by the Food and Drug Administration, tests are conducted to quantify ADAs in the subjects. Therefore, it is unlikely that patients receiving emicizumab had any clinically relevant levels of antibodies.”

Archetypes benefitting from emicizumab therapy

**Figure 3:**
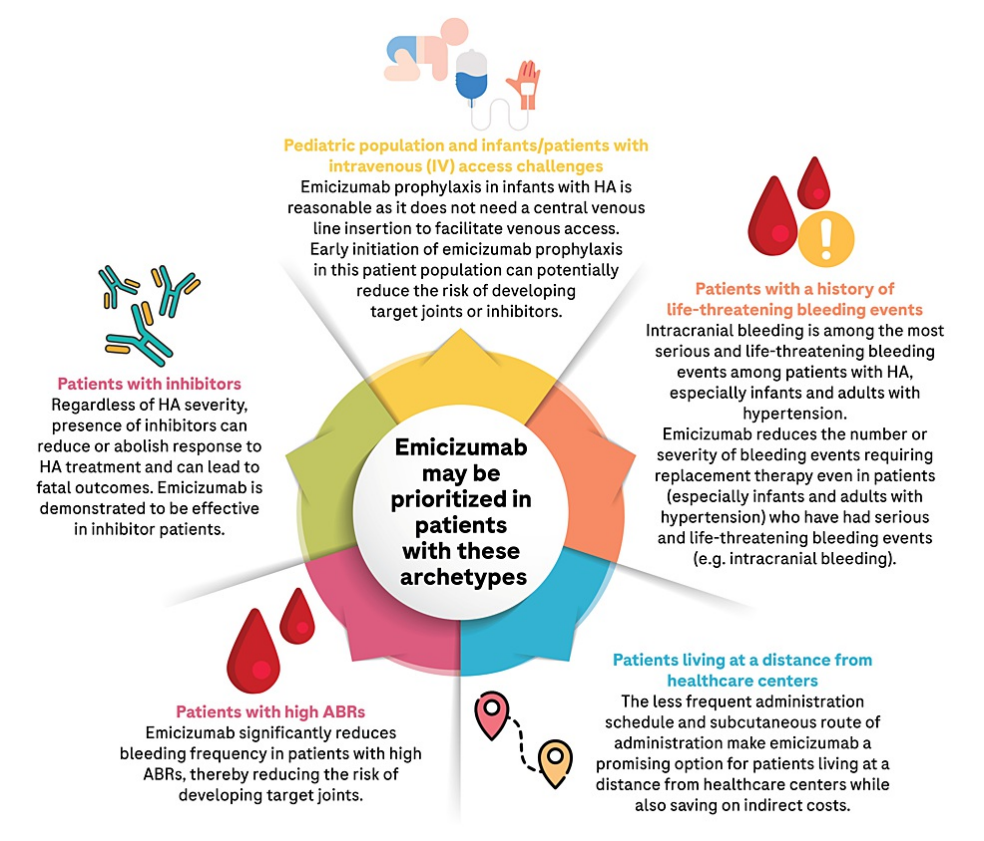
Patient archetypes in which emicizumab prophylaxis may be prioritized ABR: Annualized bleed rate; HA: Hemophilia A; ITI: Immune tolerance induction Original illustration

Although the pooled analysis from the HAVEN 1-4 trials has reported the safety and effectiveness of emicizumab prophylaxis in patients with HA across age groups and in patients with and without FVIII inhibitors [41], due to resource constraints, the use of prophylactic emicizumab over conventional therapy should be prioritized in patients with certain archetypes (Figure 3).

Patients With Inhibitors

Approximately 20%-35% of the patients with HA develop inhibitors after receiving on-demand or prophylactic FVIII-containing regimens [62]. These inhibitors neutralize the coagulation activity of exogenous FVIII and render the treatment ineffective. Emicizumab is the only choice in the management of HA patients with inhibitors. While achieving inhibitor eradication remains a desirable objective, and all patients with inhibitors should be provided with at least one opportunity for ITI, emicizumab monotherapy presents an alternative option for those who are not suitable candidates for ITI [6]. In a single-center, retrospective study on pediatric patients (N=12) with HA who transitioned to emicizumab from the previous FVIII therapy after partial or complete immune tolerization, 92% of the patients achieved negative inhibitor status at the end of the study (mean follow-up: 14.2 months) [63].

The HAVEN-1 trial compared bleeding rates among HA patients with inhibitors who previously received a bypassing agent and emicizumab prophylaxis versus patients who did not receive emicizumab prophylaxis. The results demonstrated that emicizumab prophylaxis resulted in 87% lower bleeding rates than when no prophylaxis was administered [35]. Compared to patients who received prophylaxis with a bypassing agent, emicizumab prophylaxis was associated with a 79% lower bleeding rate.

In elderly HA patients (N=12; median age: 74 years) with inhibitors (titer, 22.3 Bethesda units/mL), subcutaneous emicizumab once weekly for three weeks and then once every three weeks resulted in good hemostatic efficacy. Moreover, the patients could discontinue bypassing therapy after a median of 1.5 days [64].

The importance of follow-up, as discussed earlier, also escalates if the patients have non-severe HA but inhibitors. Data from a retrospective cohort study of 2,709 patients with non-severe HA (107 with inhibitors) indicated that the development of inhibitors in patients with non-severe hemophilia increased the mortality rate. Of the 2709 patients, 148 died, and the median age at death was 64 (49-76) years. Out of the 148 deaths, 62 (42%) deaths were hemophilia related. Among the patients with inhibitors, 16 died at a median age of 71 (60-81) years; seven of these deaths were due to severe bleeding. Inhibitor patients demonstrated >5 times the all-cause mortality rate than patients without inhibitors. Therefore, follow-up of patients with inhibitors is essential considering the high rates of deaths related to hemophilia observed in non-severe hemophilia patients with inhibitors [65].

Patients With High ABRs

Clinical trials have demonstrated that emicizumab significantly reduces the bleeding frequency in patients with HA, including those with high ABRs. Frequent bleeds can lead to significant morbidity, including joint damage, chronic pain, and decreased mobility, impacting their quality of life. Patients with high ABRs are more likely to experience severe or life-threatening bleeding events, such as intracranial hemorrhage or gastrointestinal bleeding. There is a significant correlation between high ABR and the number of target joints [66]. The median ABR, as per the 2018 report from the WFH, in patients with severe HA was 6 (range: 2-14). The highest ABR was observed in Southeast Asia (20; range: 8-30) [67]. Patients who are on on-demand therapy are reported to have 2.8-fold higher ABRs than those who received prophylactic treatment [66]. Pooled data from the HAVEN 1-4 trials reported a model-based ABR of 1.4 (1.1-1.7) for a median efficacy period of 120.4 (89.0-164.4) weeks. The mean ABR for treated bleeds reduced over successive 24-week treatment intervals, and the median ABR for treated bleeds remained 0 for the entire study period. Additionally, 97.6% of the patients on emicizumab experienced ≤3 treated bleeds, and 82.4% of patients reported zero treated events of bleeding. In the 970.3 patient-years of exposure, prophylaxis with emicizumab was associated with low bleed rates in patients with HA across age groups and in patients with and without FVIII inhibitors; therefore, emicizumab may be considered a promising therapeutic agent in patients with a high ABR [41].

Patients Living at a Distance From Healthcare Centers

A considerable distance from the patient’s homes to the treatment center might contribute to underdiagnosis and increase the risk for inhibitor development, which can add to the expenditure incurred by the patients. On average, patients in the HAEMOcare study had to travel 79.4 km (~50 miles) to reach a hemophilia care center, and the travel itself amounted to 1.4% of the monthly family income (mean: USD 907.60) [32]. However, distance alone is not an accurate measure of the burden of travel for HA patients. While greater distance implies a prolonged travel time, the relation can be disproportionate in several parts of India [68]. For example, traffic congestion and the chosen mode of transport (linked to the affordability of transport), as well as patient mobility (e.g., the orthopedic burden can limit the transport options available), can also substantially influence access to healthcare [68]. Therefore, prophylaxis-based treatment in individuals who reside far from the treatment center or need to factor in a substantial amount of travel time may help achieve better disease control, reduce joint pathology, and improve the patient’s QoL [27]. According to the WFH, patients with HA must have access to safe and effective therapeutic options with optimal efficacy in preventing bleeding and managing trauma-related, spontaneous, or breakthrough bleeding. Emicizumab is associated with the benefit of less frequent dosing of once or twice a month [10], which would reduce the frequency of travel from home to the healthcare center in patients with HA. These benefits suggest that emicizumab is a cost-effective option for HA patients who live far from a hemophilia care center [8].

Pediatric Population and Infants/Patients With Intravenous (IV) Access Challenges

It is expected that bleeding frequency can increase during infancy due to an increase in activity associated with normal growth and development of infants [69]. Prophylactic treatment at this stage appears to be reasonable. Given that emicizumab can be administered subcutaneously with longer time intervals between subcutaneous administrations, it would be an ideal primary prophylactic agent in the infant population compared to traditional agents that require frequent IV access.

A study conducted on 4- to 14-year-old children with hemophilia from Gujarat showed that the functions of standing, kneeling, and sitting were the worst affected [23]. The HAVEN 7 trial reported that, in infants with HA, early prophylaxis could reduce potentially life-threatening bleeding and long-term joint function [44]. The use of emicizumab in the management of children can substantially delay the exposure to FVIII and, thus, delay inhibitor development. Emicizumab does not need a central venous line insertion to facilitate venous access. Furthermore, emicizumab use is associated with an improved QoL and a reduced risk of intracerebral hemorrhage. The main advantages of emicizumab use in pediatric patients are subcutaneous administration, circumventing issues related to IV access, and a convenient administration frequency, improving parental adherence [56].

Patients With a History of Life-Threatening Bleeding Events

Intracranial bleeding is among the most serious and life-threatening bleeding events among patients with HA, especially infants and adults with hypertension, and it accounts for almost 20% of mortality in these patients [70]. Emicizumab has been reported to be beneficial in reducing bleeding rates even in patients who have had severe bleeding events. In the HAVEN 7 trial, no intracranial hemorrhage was reported among the 54 infants (≤12 months) who received emicizumab [71]. Emicizumab prophylaxis may attenuate the bleeding phenotype in patients with severe HA by reducing the number or severity of bleeding events requiring replacement therapy, but for patients with life-threatening bleeding, including events of intracranial bleeding or bleeding events causing disability, timely and adequate administration of FVIII is recommended even when the patient is on emicizumab prophylaxis [72].

Way forward

Innovations in the diagnosis and treatment of HA in recent years point toward a better standard of care for patients with HA. When individualizing therapy, factors such as age, bleeding phenotype, joint status, pharmacokinetics, adherence, physical activity, and personal goals should all be considered [9]. Despite regular use of diagnostic imaging and physical examinations, early detection of joint damage remains challenging. Additionally, patients on primary prophylaxis tend to develop arthropathy slowly, necessitating long-term follow-up for analysis, making it challenging to evaluate the efficacy of prophylaxis [12]. Long-term evidence on the benefits of prophylaxis with new agents such as emicizumab will change the course of clinical decision-making for HA. There is a possibility that patients on emicizumab prophylaxis may experience breakthrough bleeding events. This is particularly relevant to HA patients with inhibitors, as it would mean concomitant use of alternative hemostatic agents [73]. Evidence on the benefits of emicizumab prophylaxis in patients who are engaged in high-intensity activities could be a focus of future research.

The challenges in the management of HA in resource-limited nations (RLN) include inadequate trained manpower, limited access to diagnostic laboratories, and a lack of awareness regarding the disease among patients, family members, and the government. Although the annual WFH global survey provides absolute numbers of patients with HA in each country, it is a gross underestimate and does not truly represent the actual numbers, particularly in RLN, due to underdiagnosis. Registries may help in bridging this gap [74]. The US has a remarkable hemophilia surveillance system; the case detection rate in India is 4.7 times lower than in the US. Furthermore, studies that predict the hemophilia trends in India are limited and only gross deductions are possible from the data from Annual Global Surveys [19].

Public health agencies have a role in linking patients to necessary services. The social costs of hemophilia are due to lack of access to therapy and patient impact in terms of schooling, employment, morbidity, and mortality. Parents face financial problems due to out-of-pocket payments and experience distress in not being able to offer their children the appropriate treatment. In India, inadequate access to treatment is a major cause of the compromised QoL of patients [19].

Genetic analysis is recommended to identify carriers of HA and to facilitate genetic counseling of family members. Chorionic villous sampling in pregnant women who are carriers is usually advised at 11-14 weeks of gestation. More than 3,500 different disease-causing pathogenic variants of HA have been identified and reported in international databases such as the Factor VIII Variant Database [75]. The mutation may remain unidentified in some patients with HA on routine testing. Nevertheless, advancements in molecular diagnostics, with the introduction of next-generation sequencing/Sanger sequencing, are improving the molecular diagnostic yield. Identifying the mutation helps in preimplantation genetic diagnosis. If the fetus is affected, the family may be counseled appropriately about the diagnosis and be provided medically and legally available options for making an informed decision.

## Conclusions

Traditionally, patients with HA have been managed with CFCs that are associated with unsatisfactory compliance due to the frequent, intravenous route of administration, a short half-life, and the development of inhibitors. Emicizumab, a bispecific antibody, has shown remarkable promise in effectively controlling bleeding in HA. It has been notably positioned for use in patients with inhibitors and non-inhibitors across all age groups. It is safe and effective, improves QoL, is convenient to use, and is potentially sustainable in the long term. Despite the high cost, emicizumab is markedly cost-effective in HA patients with certain archetypes in India, such as inhibitor patients, those with high ABRs, those with poor access to a hemophilia care center, pediatric and infant populations with IV access challenges, and those with a history of life-threatening bleeding events.
