# Correction: Expert Opinions on the Management of Hemophilia A in India: The Role of Emicizumab

**DOI:** 10.7759/cureus.c172

**Published:** 2024-05-08

**Authors:** Naresh Gupta, Anupam Dutta, Bilal Ahmed, Cecil R Ross, Chandrakala S, Gerard Dolan, M J John, Nita Radhakrishnan, Sunita Aggarwal, Tulika Seth, Varun Kaul, Vijay Shah

**Affiliations:** 1 Medicine and Surgery, All India Institute of Medical Sciences, New Delhi, New Delhi, IND; 2 Haematology & Haemophilia, Maulana Azad Medical College, Lok Nayak Hospital, New Delhi, IND; 3 General Medicine, Assam Medical College and Hospital, Dibrugarh, IND; 4 Pathology, Transfusion Medicine & Hemophilia, Government Medical College, Srinagar, IND; 5 Hematology, St. John's Medical College and Hospital, Bangalore, IND; 6 Clinical Haematology, King Edward Memorial Hospital, Mumbai, IND; 7 Haematology, St. Thomas’ Haemophilia Comprehensive Care Centre, Bournemouth, GBR; 8 Clinical Hematology, Hemato-Oncology & Bone Marrow Transplant, Christian Medical College & Hospital, Ludhiana, IND; 9 Hematology and Oncology, Super Speciality Paediatric Hospital and Post Graduate Teaching Institute, Noida, IND; 10 Medicine, Maulana Azad Medical College, New Delhi, IND; 11 Hematology, All India Institute of Medical Sciences, New Delhi, New Delhi, IND; 12 Pediatrics, Guru Gobind Singh Medical College & Hospital, Faridkot, IND; 13 Pediatrics, Nirmal Hospital Pvt. Ltd., Surat, IND

This article has been corrected by the Editor-in-Chief to remove or amend four statements referring to emicizumab as an on-demand therapy. Emicizumab is not approved as an on-demand therapy, therefore the following corrections have been made to ensure scientific accuracy:

The following statements have been removed:

"On-demand emicizumab was more cost-effective than prophylactic emicizumab in non-inhibitor patients. However, in inhibitor patients, prophylactic emicizumab is cost-effective."

"Patients willing to pay only for on-demand emicizumab may lose out on its effectiveness in reducing joint disease. Until gene therapy becomes a reality, the disability caused by HA must be controlled using emicizumab.

The following statements have been revised to remove the reference to "on-demand" therapy:

"However, modeling studies have reported the cost-effectiveness of emicizumab prophylaxis over FVIII prophylaxis and over emicizumab on-demand treatment, low-dose prophylaxis, intermediate-dose prophylaxis, and high-dose prophylaxis [8,57]."

"The benefits of emicizumab override the cost of the drug. It is effective as a prophylactic, on-demand, and maintenance therapy in patients of all ages and patients with systemic diseases such as hepatic disease, and renal problems."

